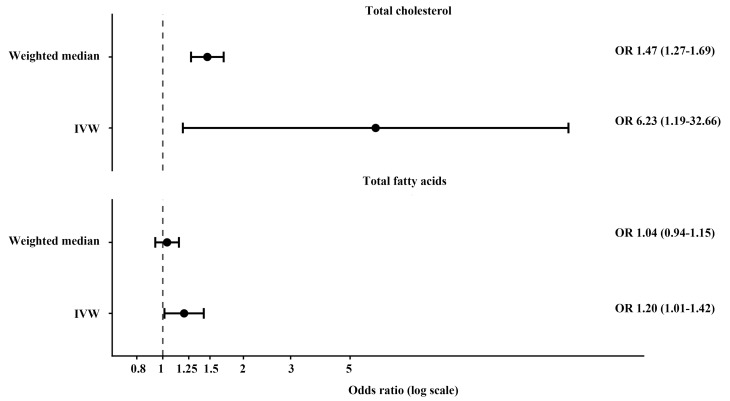# Genetically predicted stigmasterol and brain tumor risk: A Mendelian randomization study

**DOI:** 10.1097/MD.0000000000048870

**Published:** 2026-05-15

**Authors:** Binbin Zhang, Bolin Gao, Peng Yun, Shiwen Guo, Chen Liang

**Affiliations:** a Department of Neurosurgery, The First Affiliated Hospital of Xi’an Jiaotong University, Xi’an, Shaanxi, P.R. China.

**Keywords:** brain tumor, cholesterol, genetic epidemiology, lipid metabolism, Mendelian randomization, stigmasterol

## Abstract

Stigmasterol, a plant-derived sterol involved in lipid metabolism, has been implicated in cancer-related biological processes. However, its causal relevance to brain tumor risk remains unclear. This study aimed to assess the causal relationships among genetically predicted stigmasterol levels, lipid metabolic traits, and brain tumor risk using Mendelian randomization (MR). Two-sample MR analysis was conducted using summary statistics from large-scale genome-wide association studies of European ancestry. Genetically predicted stigmasterol levels were used as the exposure, lipid metabolic traits as secondary exposures, and brain tumor risk as the outcome. Sensitivity analyses included MR-Egger, weighted median, heterogeneity tests, and leave-one-out analyses. Multivariable MR analysis was performed to evaluate the independent effects of stigmasterol and total cholesterol. Genetically predicted stigmasterol levels were not significantly associated with overall brain tumor risk (OR = 1.14, 95% CI 0.49–2.63), with consistent null findings across sensitivity analyses and analyses restricted to glioblastoma. In contrast, genetically predicted stigmasterol levels were associated with increased total cholesterol (odds ratio [OR] = 6.23, 95% confidence interval [CI] 1.19–32.66) and fatty acid levels (OR = 1.30, 95% CI 1.08–1.57). Total cholesterol showed suggestive and method-dependent evidence of an association with brain tumor risk in the weighted median analysis, whereas total fatty acids showed no consistent association. In multivariable MR analyses, neither stigmasterol nor total cholesterol showed an independent association with brain tumor risk. This MR study does not support a strong direct causal effect of genetically predicted stigmasterol on brain tumor risk. However, its close genetic association with lipid metabolism highlights lipid metabolic pathways as a relevant context for understanding brain tumor susceptibility.

## 1. Introduction

Brain tumors constitute a heterogeneous group of malignancies characterized by aggressive biological behavior and poor clinical outcomes, particularly in high-grade subtypes such as glioblastoma.^[1]^ Despite advances in surgical resection, radiotherapy, and systemic therapies, prognosis remains unfavorable for many patients, underscoring the need to better understand etiological mechanisms underlying brain tumor development and progression.^[1]^ Increasing evidence suggests that metabolic reprogramming is a fundamental hallmark of brain tumors, enabling malignant cells to sustain rapid proliferation, resist therapy, and adapt to hostile microenvironments.^[2,3]^

Among metabolic pathways, lipid metabolism has emerged as a critical regulator of tumor biology.^[4]^ Alterations in cholesterol homeostasis, fatty acid synthesis, and lipid-mediated signaling have been implicated in tumor growth, immune modulation, and therapeutic resistance in brain tumors.^[4]^ Observational studies have reported associations between circulating lipid levels and brain tumor risk; however, these findings are vulnerable to residual confounding, reverse causation, and measurement bias.^[5,6]^ Consequently, whether lipid metabolic traits exert a causal influence on brain tumor susceptibility remains incompletely understood.

Stigmasterol is a major plant-derived sterol structurally similar to cholesterol and widely present in the human diet.^[7]^ Experimental studies have suggested that stigmasterol may influence lipid metabolism, inflammatory signaling, oxidative stress, and cell proliferation.^[7,8]^ In cancer research, stigmasterol has been reported to exert antitumor effects in multiple malignancies, primarily based on in vitro experiments and animal models.^[7,8]^ However, evidence regarding its role in human brain tumors is limited.^[8–10]^ Importantly, no prior investigation has systematically evaluated whether circulating stigmasterol has a causal effect on brain tumor risk at the population level, nor whether such effects may be mediated through lipid metabolic pathways.

Observational studies examining associations between metabolic factors and cancer risk are inherently susceptible to residual confounding and reverse causation, particularly in the context of diet-related exposures and complex metabolic traits.^[11]^ Mendelian randomization (MR) is a genetic epidemiological approach that uses germline genetic variants as instrumental variables to strengthen causal inference by approximating the random allocation of exposures at conception. Because genetic variants are fixed prior to disease onset and are largely independent of environmental and behavioral confounders, MR provides a robust framework for evaluating the potential causal relevance of circulating metabolic factors and for disentangling complex exposure–disease relationships.^[12,13]^

To address these gaps, we applied a comprehensive MR framework to investigate the causal relationships among genetically predicted stigmasterol levels, lipid metabolic traits, and brain tumor risk. Specifically, this study aimed to assess the causal association between stigmasterol and brain tumor risk; evaluate the effects of stigmasterol on major lipid metabolic traits; and explore whether lipid metabolic traits account for the association between stigmasterol and brain tumor susceptibility and evaluate their independent effects using multivariable Mendelian randomization (MVMR). By integrating genetic evidence across multiple metabolic layers, this study provides a genetically informed perspective on the role of plant sterols and lipid metabolism in brain tumor biology.

## 2. Methods

### 2.1. Study design

This study was conducted as a 2-sample MR analysis to evaluate potential causal relationships among stigmasterol, lipid metabolic traits, and brain tumor risk. The study was not preregistered and relied on the covariate adjustments implemented in the original GWAS consortia, as described in the corresponding GWAS reports. MR leverages genetic variants as instrumental variables (IVs) to strengthen causal inference by minimizing confounding and reverse causation. The validity of MR relies on 3 core assumptions: the genetic instruments are robustly associated with the exposure of interest; the instruments are not associated with confounders of the exposure–outcome relationship; and the instruments influence the outcome exclusively through the exposure.

**Figure 1.**
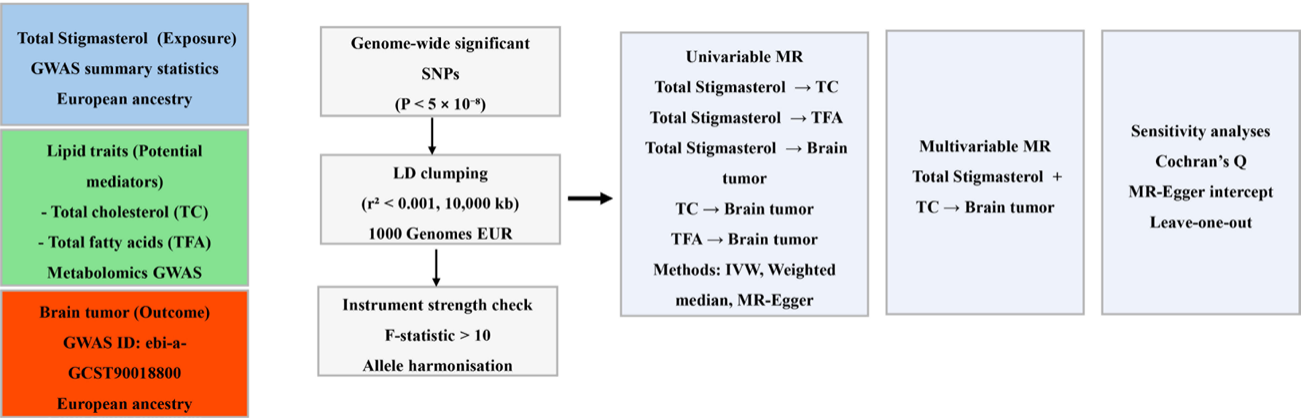
Schematic overview of the study design and analytical workflow of the Mendelian randomization analyses. GWAS = genome-wide association studies, IVW = inverse-variance weighted, LD = linkage disequilibrium, MVMR = multivariable Mendelian randomization, MR = Mendelian randomization, SNP = single-nucleotide polymorphism.

The analytical framework first examined the association between genetically predicted stigmasterol levels and brain tumor risk. Subsequently, the effects of stigmasterol on lipid metabolic traits were evaluated, followed by analyses of the associations between lipid traits and brain tumor risk. Finally, MVMR was applied to estimate the independent effects of stigmasterol and lipid metabolic traits on brain tumor risk (Fig. 1).

### 2.2. Data sources

Genome-wide association study (GWAS) summary statistics for circulating stigmasterol levels were obtained from large-scale metabolomic GWASs conducted in populations of European ancestry.^[14]^ Summary-level GWAS data for brain tumors, including overall brain tumors and glioblastoma, were derived from publicly available consortia and accessed via the OpenGWAS database.^[15]^ GWAS summary statistics for lipid metabolic traits, including total cholesterol (TC) and total fatty acids (TFA), were obtained from large population-based studies available through the same platform. A summary of data sources for exposures, outcomes, and sample sizes is provided in Table 1. To minimize potential bias due to sample overlap, the GWASs used for exposures and outcomes were derived from independent or minimally overlapping cohorts, all restricted to individuals of European ancestry. Any residual overlap was considered unlikely to materially influence the causal estimates given the strength of the genetic instruments. All analyses were based on publicly available summary-level GWAS data; therefore, no additional ethical approval, participant consent, or individual-level eligibility criteria were required. Statistical power was primarily determined by the sample sizes of the original GWAS consortia.

**Table 1:** Summary of data sources for exposures, outcomes, and sample sizes.

Trait	Role in MR	Data source	GWAS ID/Accession	Ancestry	Sample size (N)
Stigmasterol	Primary exposure	Genome-wide phytosterol GWAS meta-analysis (Nat Commun, 2022)	DOI: 10.1038/s41467-021-27706-6	European	9758
Brain tumor	Primary outcome	IEU OpenGWAS dataset	ebi-a-GCST90018800	European	491,542
Total cholesterol (TC)	Secondary exposure and MVMR covariate	IEU OpenGWAS dataset	ebi-a-GCST90025953	European	437,878
Total fatty acids (TFA)	Secondary exposure	IEU OpenGWAS dataset	met-d-Total_FA	European	114,999
Glioblastoma (GBM)	Secondary outcome	IEU OpenGWAS dataset	finn-b-C3_GBM	European	218,792

GWAS = genome-wide association studies, IEU = integrative epidemiology unit, MR = mendelian randomization, MVMR = multivariable Mendelian randomization.

### 2.3. Data harmonization

Single-nucleotide polymorphism (SNP) harmonization was performed to align effect alleles between exposure and outcome datasets. Palindromic SNPs with ambiguous allele frequencies were excluded to minimize strand ambiguity.

### 2.4. Selection of genetic instruments

SNPs associated with stigmasterol at genome-wide significance (*P* < 5 × 10^−8^) were selected as IVs. Linkage disequilibrium clumping was applied using an *r*^2^ threshold of 0.001 within a 10,000 kb window to ensure independence among instruments. Instrument strength was assessed using *F*-statistics, with values exceeding the conventional threshold (*F* > 10) indicating a low risk of weak instrument bias.

### 2.5. Mendelian randomization analyses

The inverse-variance weighted (IVW) method was used as the primary MR estimator. Weighted median and MR-Egger regression were employed as complementary approaches to assess the robustness of causal estimates under different assumptions regarding horizontal pleiotropy. Between-variant heterogeneity was evaluated using Cochran Q statistic, and directional pleiotropy was assessed using the MR-Egger intercept. Leave-one-out and funnel plot analyses were conducted to examine the influence of individual SNPs on overall causal estimates. Sensitivity analyses included MR-Egger regression, weighted median estimation, Cochran Q test for heterogeneity, MR-Egger intercept test for directional pleiotropy, and leave-one-out analyses. Effect estimates are reported as odds ratios (ORs) per genetically predicted unit increase in the exposure, corresponding to 1 standard deviation increase in circulating stigmasterol or lipid traits, unless otherwise specified. Secondary analyses were conducted using glioblastoma (GBM) as the outcome. Given the prespecified primary analysis, secondary and exploratory analyses were interpreted cautiously, and no formal multiple-testing correction was applied.

### 2.6. Multivariable Mendelian randomization (MVMR)

MVMR was performed by simultaneously modeling genetically predicted stigmasterol and total cholesterol as exposures. This approach enables estimation of their independent effects on brain tumor risk while accounting for correlations between exposures and shared genetic architecture.

### 2.7. Statistical software

All statistical analyses were conducted using R software (version 4.5.1). Two-sample MR analyses were performed using the TwoSampleMR package (version 0.6.29). Multivariable MR analyses were implemented using a multivariable inverse-variance weighted approach. Figures were generated using the ggplot2 package (version 4.0.1).

### 2.8. Ethics statement

As all data were derived from publicly available GWAS summary statistics, no additional ethical approval or informed consent was required.

## 3. Results

### 3.1. Genetic instruments for stigmasterol

A total of 15 independent SNPs strongly associated with stigmasterol were selected as IVs. All instruments demonstrated sufficient strength, with *F*-statistics well above the conventional threshold (see Table, Supplemental Digital Content 1, which summarizes the instrumental variables), indicating a low risk of weak instrument bias. Harmonization procedures retained the majority of SNPs for downstream analyses, ensuring consistency across exposure and outcome datasets (see Table, Supplemental Digital Content 2, which shows details of data harmonization procedures and retained variants).

### 3.2. Genetically predicted stigmasterol and brain tumor risk

**Figure 2.**
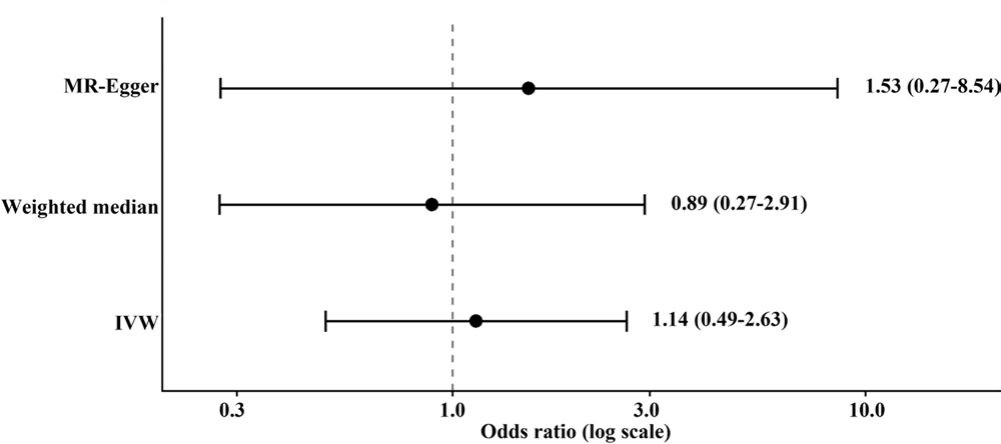
Genetically predicted total stigmasterol and risk of brain tumor. IVW = inverse-variance weighted, MR = Mendelian randomization.

Using the IVW method, genetically predicted stigmasterol levels were not significantly associated with overall brain tumor risk (OR = 1.14, 95% confidence interval [CI] 0.49–2.63, *P* = .763). Consistent null associations were observed using the weighted median (OR = 0.89, 95% CI 0.27–2.91) and MR-Egger methods (OR = 1.53, 95% CI 0.27–8.54), with effect estimates spanning both sides of the null. These results are summarized in Table 2 (Panel A) and visualized in Figure 2.

**Table 2:** Univariable Mendelian randomization analyses of stigmasterol, lipid traits, and brain tumor risk.

Panel A. Stigmasterol → Brain tumor									
Exposure	Outcome	Method	N SNPs	Beta	SE	*P* value	OR (95% CI)	Q *P* value	Egger intercept *P*
Total stigmasterol	Brain tumor	IVW	15	0.129	0.428	.763	1.14 (0.49–2.63)	.825	.707
Total stigmasterol	Brain tumor	Weighted median	15	-0.117	0.580	.841	0.89 (0.27–2.91)		
Panel B. Stigmasterol → Lipid traits									
Exposure	Outcome	Method	N SNPs	Beta	SE	*P* value	OR (95% CI)	Q *P* value	Egger intercept *P*
Total stigmasterol	Total cholesterol	IVW	7	1.829	0.845	.030	6.23 (1.19–32.66)	.056	.315
Total stigmasterol	Total cholesterol	Weighted median	7	0.383	0.072	8.6 × 10^−8^	1.47 (1.27–1.69)		
Total stigmasterol	Total fatty acids	IVW	17	0.262	0.095	.006	1.30 (1.08–1.57)	.132	.961
Total stigmasterol	Total fatty acids	Weighted median	17	0.110	0.062	.075	1.26 (0.90–1.77)		
Panel C. Lipid traits → Brain tumor									
Exposure	Outcome	Method	N SNPs	Beta	SE	*P* value	OR (95% CI)	Q *P* value	Egger intercept *P*
Total cholesterol	Brain tumor	IVW	192	0.160	0.117	.171	1.17 (0.93–1.47)	.055	.614
Total cholesterol	Brain tumor	Weighted median	192	0.370	0.185	.046	1.45 (1.01–2.08)		
Total fatty acids	Brain tumor	IVW	58	-0.156	0.118	.188	0.86 (0.68–1.08)	.403	.440
Total fatty acids	Brain tumor	Weighted median	58	-0.319	0.186	.087	0.73 (0.50–1.05)		

CI = confidence interval; Q, Cochran Q statistic, GWAS = genome-wide association studies, IVW = inverse-variance weighted, OR = odds ratio, SE = standard error, SNP = single-nucleotide polymorphism.

**Figure 3.**
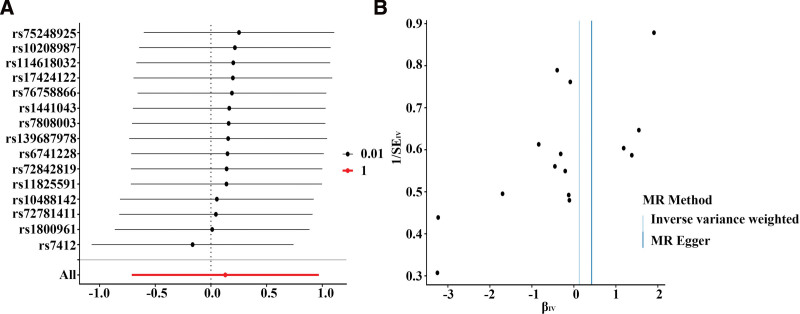
Sensitivity analyses of the causal association between genetically predicted total stigmasterol and brain tumor risk. (A) Leave-one-out analysis; (B) Funnel plot. IV = instrumental variable, MR = Mendelian randomization, SE = standard error.

Sensitivity analyses indicated no substantial heterogeneity or evidence of directional pleiotropy (see Table, Supplemental Digital Content 3, which reports heterogeneity and pleiotropy statistics). Leave-one-out and funnel plot analyses further confirmed that no single genetic variant disproportionately influenced the overall causal estimates (Figure 3; see Figure, Supplemental Digital Content 4, which presents SNP-level scatter plots, and Figure, Supplemental Digital Content 5, which shows SNP-specific MR estimates).

### 3.3. Genetically predicted stigmasterol and lipid metabolic traits

In contrast to the null association with brain tumor risk, genetically predicted stigmasterol levels were robustly associated with lipid metabolism–related traits. Higher genetically predicted stigmasterol levels were associated with increased TC using both the IVW method (OR = 6.23, 95% CI 1.19–32.66, *P* = .030) and the weighted median method (OR = 1.47, 95% CI 1.27–1.69, *P *= 8.6 × 10^−8^), with no evidence of directional pleiotropy.

Similarly, genetically predicted stigmasterol levels were positively associated with total fatty acids in the IVW analysis (OR = 1.30, 95% CI 1.08–1.57, *P* = .006). Although the weighted median estimate showed a consistent direction of effect, statistical significance was not retained. Detailed results are presented in Table 2 (Panel B), with additional lipid traits reported in the supplementary materials (see Figure, Supplemental Digital Content 6, which shows additional MR results for lipid traits and Table, Supplemental Digital Content 7, which shows detailed results for additional lipid metabolic traits).

### 3.4. Lipid metabolic traits and brain tumor risk

Subsequent MR analyses evaluated the causal relationships between lipid metabolic traits and brain tumor risk. Genetically predicted TC demonstrated suggestive evidence of an association with brain tumor risk, achieving statistical significance in the weighted median analysis (OR = 1.45, 95% CI 1.01–2.08, *P* = .046) but not in the primary IVW analysis. In contrast, no consistent evidence supported a causal association between TFA and brain tumor risk across MR methods. These results are summarized in Table 2 (Panel C).

### 3.5. MVMR analysis

To assess whether stigmasterol influences brain tumor risk independently of lipid metabolism, multivariable MR analyses were conducted including stigmasterol and total cholesterol as simultaneous exposures. In this model, neither genetically predicted stigmasterol (OR = 0.76, 95% CI 0.25–2.33, *P* = .631) nor TC (OR = 1.22, 95% CI 0.88–1.68, *P* = .240) showed a statistically significant independent association with brain tumor risk (Table 3).

**Table 3:** Multivariable Mendelian randomization analysis results.

Outcome	Exposure	N SNPs	Beta	SE	*P* value	OR (95% CI)
Brain tumor	Total stigmasterol	172	-0.275	0.573	.631	0.76 (0.25–2.33)
Brain tumor	Total cholesterol	172	0.195	0.165	.240	1.22 (0.88–1.68)

CI = confidence interval, OR = odds ratio, SE = standard error, SNP = single-nucleotide polymorphism.

### 3.6. Secondary analyses using GBM as outcome

Secondary MR analyses using GBM as a specific brain tumor subtype yielded no significant associations for stigmasterol, TC, or TFA. These findings were consistent with the primary analyses (see Table, Supplemental Digital Content 8, which presents the results of secondary analyses using glioblastoma as the outcome).

## 4. Discussion

This study applied a comprehensive MR framework to investigate the causal architecture linking stigmasterol, lipid metabolism, and brain tumor susceptibility. The principal finding was the absence of strong evidence supporting a direct causal effect of genetically predicted stigmasterol on brain tumor risk. This null association was consistent across multiple MR estimators, sensitivity analyses, and secondary analyses focusing on glioblastoma. In contrast, genetically predicted stigmasterol demonstrated a strong causal association with TC and a weaker or less consistent association with TFAs. Furthermore, TC showed suggestive evidence of an association with brain tumor risk, whereas no consistent evidence of association was observed for TFA.

Experimental studies have long suggested potential antitumor effects of stigmasterol, including inhibition of proliferation, induction of apoptosis, and modulation of inflammatory and oxidative stress pathways.^[7,8]^ More recently, in vitro studies have reported that stigmasterol suppresses glioma and glioblastoma cell growth, potentially through lipid metabolism–related mechanisms.^[9,10]^ However, the present MR findings should be interpreted in light of fundamental differences between experimental and genetic epidemiological approaches. Experimental models typically involve short-term exposure to supraphysiological concentrations of stigmasterol, whereas MR captures the effect of lifelong genetically proxied variation in circulating levels. The absence of a detectable direct causal effect in the MR setting therefore does not contradict experimental observations but instead suggests that such effects may be context-dependent and insufficient to influence population-level disease incidence.

Several biological considerations may explain the null association observed. First, lipid metabolism in the central nervous system is uniquely regulated, with the brain relying largely on de novo cholesterol synthesis and minimal contribution from circulating sterols due to the blood–brain barrier.^[16,17]^ Consequently, genetically determined variation in circulating stigmasterol may have limited direct impact on the intracerebral lipid environment relevant to tumor initiation or early susceptibility. Second, brain tumors are highly heterogeneous with respect to molecular drivers and metabolic dependencies.^[18,19]^ If stigmasterol influences only specific subtypes or stages of tumor development, such effects may be diluted in GWAS-based MR analyses evaluating overall disease risk.

A central contribution of this study lies in contextualizing stigmasterol within a broader lipid metabolic framework. The robust associations observed between genetically predicted stigmasterol and lipid traits are consistent with the established genetic coupling between plant sterols and systemic lipid homeostasis.^[20,21]^ Notably, the positive associations between stigmasterol and TC observed in the MR analyses differ from cholesterol-lowering effects reported in short-term dietary intervention studies.^[22,23]^ This apparent discrepancy is biologically and methodologically plausible and has also been highlighted in recent large-scale genetic studies of phytosterols. A genome-wide meta-analysis of phytosterols demonstrated that genetically elevated circulating phytosterol levels, including sitosterol, were associated with adverse cardiovascular outcomes, with evidence that these effects were partially mediated through cholesterol-related pathways.^[14]^ That study further revealed that genetic determinants of circulating phytosterols map to key genes involved in sterol absorption, synthesis, transport, and clearance, such as *NPC1L1, HMGCR, SCARB1*, and *ABCG5/8*, underscoring the tight genetic coupling between plant sterols and systemic lipid metabolism. Although genetically predicted stigmasterol was also associated with TFA, this relationship appeared less specific and may reflect broader lipid metabolic coupling rather than a distinct metabolic pathway directly relevant to disease susceptibility. Taken together, these findings suggest that genetically predicted stigmasterol reflects a lifelong perturbation of systemic lipid metabolism rather than the short-term cholesterol-lowering effects observed in dietary intervention studies, thereby reconciling the apparent discrepancy between genetic and nutritional evidence.

The suggestive and method-dependent association between TC and brain tumor risk further supports lipid metabolism as a biologically relevant axis in brain tumor biology.^[24]^ Cholesterol plays a central role in membrane biogenesis, lipid raft formation, and intracellular signaling, all of which are critical for rapid cell proliferation and oncogenic signaling.^[25,26]^ In the central nervous system, cholesterol homeostasis is tightly regulated, and dysregulated cholesterol synthesis, trafficking, or storage has been increasingly recognized as a hallmark of malignant gliomas.^[4,27,28]^ Experimental studies have shown that glioblastoma cells exhibit enhanced reliance on cholesterol biosynthesis and uptake to sustain membrane remodeling, energy demands, and resistance to therapeutic stress, highlighting cholesterol metabolism as a metabolic vulnerability in this tumor type.^[27,28]^ Within this context, the present findings support a model in which lipid metabolism represents a biologically relevant pathway linking stigmasterol-related genetic variation to brain tumor susceptibility, even in the absence of a direct causal effect of stigmasterol itself. Rather than acting as an independent determinant of tumor risk, stigmasterol-associated genetic variation may reflect broader perturbations in systemic lipid metabolic regulation, which in turn shape a metabolic milieu permissive to tumor initiation or progression.

The MVMR analysis further refines this interpretation. MVMR extends the conventional MR framework by simultaneously modeling multiple related exposures, allowing estimation of their independent effects on an outcome while accounting for shared genetic architecture.^[29]^ This approach is particularly useful when exposures are biologically correlated, as it helps disentangle whether observed associations reflect direct causal effects or arise from overlapping metabolic pathways.^[30]^ When stigmasterol and TC were modeled simultaneously, neither exposure retained a statistically significant independent association with brain tumor risk. This finding should not be interpreted as evidence against the relevance of lipid metabolism; rather, it suggests that the effects of stigmasterol and cholesterol may be distributed across shared or correlated metabolic pathways, highlighting the complexity of lipid metabolic pathways and shared genetic architecture.

In light of the attenuation of associations observed in the multivariable MR analyses, several explanations merit consideration. First, stigmasterol may influence brain tumor risk indirectly through modulation of lipid traits that are not fully captured by TC alone, such as lipoprotein composition, fatty acid saturation patterns, or intracellular lipid flux. Second, shared genetic instruments and pathway overlap may reduce the ability of MVMR to disentangle independent effects when exposures are biologically intertwined. Finally, the relevant causal biology may operate downstream of the measured exposures, highlighting the need for more granular lipidomic and tissue-specific data in future studies.

Taken together, these findings shift the focus from a simplistic exposure–outcome relationship toward a pathway-oriented understanding of stigmasterol biology. Rather than acting as a direct determinant of brain tumor risk, stigmasterol appears to function as a component of systemic lipid metabolic regulation, which in turn may shape a metabolic milieu permissive to tumor development.

Future studies could extend this work by integrating brain-specific lipidomic quantitative trait loci, cerebrospinal fluid biomarkers, or colocalization analyses to refine tissue-specific causal inference. Additionally, combining MR findings with experimental models that interrogate lipid metabolic vulnerabilities in glioma could help bridge the gap between genetic evidence and cellular mechanisms.

This study benefits from a rigorous MR design, the application of complementary estimators, extensive sensitivity analyses, and a multivariable framework that collectively strengthen causal interpretation. Nevertheless, several limitations should be acknowledged. Although multiple sensitivity analyses were conducted to assess potential violations of MR assumptions, including tests for heterogeneity and horizontal pleiotropy, residual pleiotropic effects cannot be completely ruled out for complex metabolic traits. Moreover, genetically proxied stigmasterol reflects circulating concentrations rather than intracerebral exposure, which may limit direct inference regarding brain-specific biological processes. Statistical power may also be constrained for rare tumor subtypes, and the use of aggregate lipid traits could obscure more nuanced metabolic mechanisms operating at the cellular or tissue level. Accordingly, as with all MR studies, the present findings should be interpreted as evidence of genetic causal inference rather than direct clinical or interventional effects.

## 5. Conclusion

In conclusion, the present MR evidence does not support a strong direct causal effect of genetically predicted stigmasterol on brain tumor risk. However, stigmasterol demonstrates robust genetic associations with lipid metabolic traits, particularly TC. Notably, only TC showed suggestive evidence of an association with brain tumor susceptibility. These findings underscore lipid metabolism as a biologically relevant axis linking plant sterols to brain tumor biology and provide a genetically informed framework for future mechanistic and translational investigations.

## Acknowledgments

The authors acknowledge the use of ChatGPT, a generative artificial intelligence tool developed by OpenAI, to assist in drafting and refining the manuscript. ChatGPT was used for improving the clarity and flow of the manuscript’s text. The authors take full responsibility for the content of the manuscript, and ensure that no breach of publication ethics occurred in the process.

## Author contributions

**Conceptualization:** Shiwen Guo, Chen Liang.

**Data curation:** Binbin Zhang, Bolin Gao.

**Formal analysis:** Binbin Zhang, Bolin Gao.

**Methodology:** Shiwen Guo, Chen Liang.

**Visualization:** Peng Yun.

**Writing – original draft:** Binbin Zhang, Chen Liang.

**Writing – review & editing:** Binbin Zhang, Bolin Gao, Peng Yun, Shiwen Guo, Chen Liang.











**Figure:**
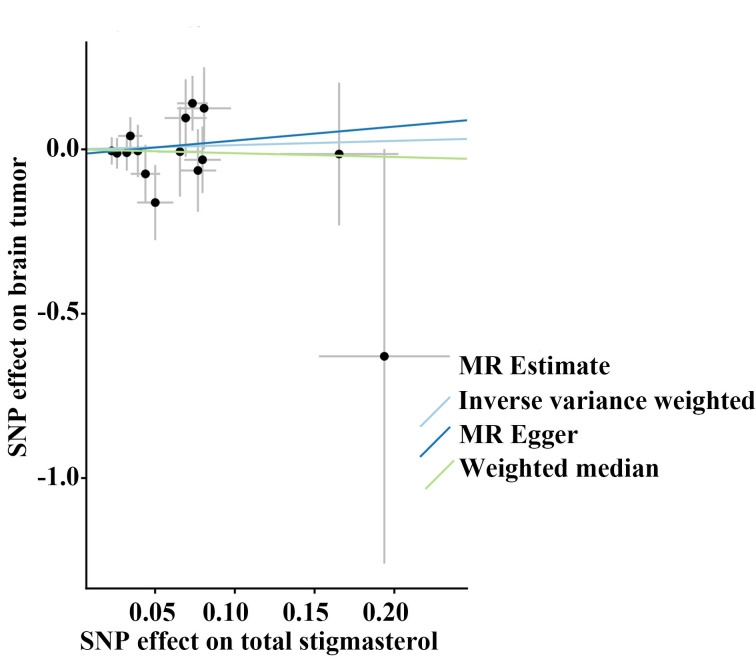


**Figure:**
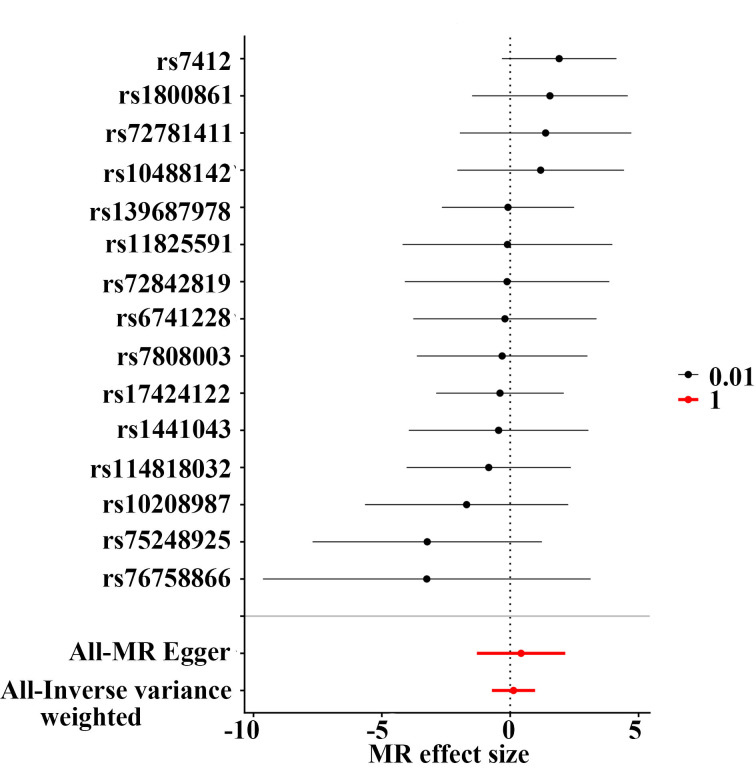


**Figure:**